# Preoperative Use and Discontinuation of Traditional Chinese Herbal Medicine and Dietary Supplements in Taiwan: A Cross-Sectional Questionnaire Survey

**DOI:** 10.3390/healthcare11111605

**Published:** 2023-05-30

**Authors:** Te-Chun Yeh, Shung-Tai Ho, Che-Hao Hsu, Ju-O Wang, Senyeong Kao, Yi-Chang Su, Sunny Jui-Shan Lin, Huei-Han Liou, Tso-Chou Lin

**Affiliations:** 1 Development and Planning Center, Taipei City Hospital, Taipei 10341, Taiwan; chun0417@gmail.com; 2 Department of Anesthesiology, Kaohsiung Medical University Hospital, Kaohsiung Medical University, Kaohsiung 80756, Taiwan; 1070091@ms.kmuh.org.tw; 3 Department of Anesthesiology, Tri-Service General Hospital, National Defense Medical Center, Taipei 11490, Taiwan; 4 Department of Anesthesiology, Tungs’ Taichung MetroHarbor Hospital, Taichung 43503, Taiwan; t11772@ms.sltung.com.tw; 5 School of Public Health, National Defense Medical Center, Taipei 11490, Taiwan; 105082@mail.ndmctsgh.edu.tw (J.-O.W.); kao@mail.ndmctsgh.edu.tw (S.K.); 6 National Research Institute of Chinese Medicine, Ministry of Health and Welfare, Taipei 11221, Taiwan; sychang@nricm.edu.tw; 7 Department of Chinese Medicine, Tri-Service General Hospital, National Defense Medical Center, Taipei 11490, Taiwan; 8 Department of Medical Education and Research, Kaohsiung Veterans General Hospital, Kaohsiung 81362, Taiwan; slove0726@hotmail.com

**Keywords:** traditional Chinese herbal medicine, dietary supplements, preoperative, discontinuation

## Abstract

Traditional Chinese herbal medicine has widespread use in Taiwan. This cross-sectional questionnaire survey investigates the preoperative use and discontinuation of Chinese herbal medicine and dietary supplements among Taiwanese patients. We obtained the types, frequency, and sources of Chinese herbal remedies and supplements used. Among 1428 presurgical patients, 727 (50.9%) and 977 (68.4%) reported the use of traditional Chinese herbal medicine and supplements in the past one month, respectively. Only 17.5% of the 727 patients stated discontinuation of herbal remedies 4.7 ± 5.1 (1–24) days before the surgery, and 36.2% took traditional Chinese herbal medicine with concomitant physician-prescribed Western medicine for their underlying diseases. The most commonly used Chinese herbs are goji berry (*Lycium barbarum*) (62.9%) and Si-Shen-Tang (48.1%) in single and compound forms, respectively. The presurgical use of traditional Chinese herbal medicine was common in patients undergoing gynecologic (68.6%) surgery or diagnosed with asthma (60.8%). Women and those with a high household income had a greater tendency to use herbal remedies. This study demonstrates the high proportion of the presurgical use of Chinese herbal remedies and supplements along with physician-prescribed Western medicine in Taiwan. Surgeons and anesthesiologists should be aware of the potential adverse effects of drug–herb interaction for Chinese patients.

## 1. Introduction

In Asian countries, traditional Chinese medicine has a long history of use in disease prevention and treatment and as daily food ingredients to increase longevity. In recent decades, herbal medicine has gained an increasing popularity in Western societies [1], for example, in opioid-sparing pain management [2]. However, since 2001, some concerns have been raised regarding herbal remedies in terms of perioperative herb–drug interactions and organ dysfunction [3,4,5,6,7], potentially toxic ingredients and heavy metal contaminants [8], and the detrimental effects of dual immunomodulation on allergic and autoimmune diseases [9]. Female patients with reproductive problems, particularly those undergoing a gynecologic procedure, tended to use herbal medicine [10], and those who used certain anticoagulant botanicals close to the surgery date had a higher risk of blood loss during the surgery [11].

The current use of herbal medicines should be specifically included in preoperative assessments. Preoperative surveys have revealed the prevalence of herbal medicine usage before admission to be 4.8% in the UK [12], 16% in New York [10], 3.6% in Hungary [13], and 14.5% in Turkey [14]. In addition, 8%–13% of surgical patients in Hong Kong used traditional Chinese herbal medicine (TCHM) preoperatively [15,16]. The American Society of Anesthesiologists recommends that patients using herbal and dietary supplements must inform their anesthesiologists regarding their use and, in some cases, must stop taking the supplements at least 2 weeks before a surgical procedure [17]. Nevertheless, patients are often unaware of this recommendation, and furthermore, they may present for emergency surgery.

In Taiwan, up to 28.4% of the 21 million population in 2001 ever received traditional Chinese medicine under the comprehensive coverage of Taiwan National Health Insurance [18], which included a large majority of Chinese herbal remedies (85.9%). Additionally, the 2005 Taiwan National Health Interview Survey revealed that 5.2% of Taiwanese adults self-administered TCHM without an appropriate prescription [19]. In a hospital-based survey, traditional Chinese medicine physicians were found to prescribe 4.87 Chinese herbal medicines on average per prescription for outpatients of the Traditional Medicine Department of a conventional Western medical center in Taiwan [20]. However, the prevalence of preoperative TCHM usage among surgical patients remains hitherto unclear in Taiwan. Additionally, TCHMs used in Chinese societies is different from herbal medicines commonly used in Western countries [5,6]. Hence, we conducted a questionnaire survey to investigate the prevalence of the preoperative use of TCHMs and dietary supplements in terms of the type, source, frequency, concomitant conventional Western medication, discontinuation, and predisposing factors among surgical patients in Taiwan.

## 2. Materials and Methods

### 2.1. Participants and Study Design

After approval was obtained from the Institutional Review Board of Tri-Service General Hospital (TSGHIRB-097-05-142-A), adult patients undergoing elective surgery were recruited from April 2009 to January 2011. Patients who could not read or fill out the Chinese questionnaire and non-Chinese speakers were excluded. After signing the written informed consent form, participants filled the questionnaires themselves or with verbal help from a trained research assistant on the day before the operation.

### 2.2. Study Instrument

The Chinese language questionnaire was largely based on prior similar surveys [9,11,14,15,19], but to increase content validity, it was refined by the review committee of seven senior specialists with expertise in traditional Chinese medicine and questionnaire surveys, which consisted of two traditional Chinese medicine physicians, two anesthesiologists, one surgeon, one pharmacologist, and one epidemiologist. To examine test–retest reliability, 18 participants in Taipei, Taiwan were asked to complete a follow-up questionnaire 2–3 weeks after completion of the first questionnaire. The overall consistency of the retest was 90.7%. Test–retest reliabilities were evaluated through the calculation of the intraclass correlation coefficient for each item in scales, with acceptable values of ≥0.55. Internal consistencies were estimated through the computation of the internal consistency coefficients of the Cronbach’s alpha, with the acceptable value being ≥0.70.

The first section of the questionnaire included the patient characteristics; diagnoses for surgical procedures; comorbidities, such as hypertension, diabetes, and asthma; and medications before admission. The second section contained a list of 158 commonly used items of traditional Chinese herbal medications and supplements, including 43 Chinese herbal compound preparations, 44 single Chinese herbs, and 71 supplements in Chinese language.

### 2.3. Data Collection

Participants were asked to indicate the types of remedies and supplements used; sources of traditional Chinese medicine, that is, from a doctor or Chinese herbal medicine store; and the frequency of medicine use before their hospitalization in the past 1 month, as well as how many days prior to surgery the medicine was discontinued.

### 2.4. Statistical Analysis

All values are expressed as mean ± standard deviation or number (percent). The chi-square test and multivariate logistic regression were used to identify predisposing factors for the use of herbal remedies and supplements before the operation. A *p* value of <0.05 was considered statistically significant. All data analyses were performed using SPSS version 22 (IBM, Armonk, NY, USA).

## 3. Results

### 3.1. General Characteristics of Participants

Among the 1958 presurgical patients asked to participate, 1428 (72.9%) (623 women and 805 men) completed the questionnaires. The demographic data are summarized in Table 1. Among the 1428 patients, 727 (50.9%) reported frequent or occasional use of TCHM and 977 (68.4%) took supplements before admission, whereas 612 (42.9%) patients took TCHM and supplements concurrently. Among the 727 patients, 329 (53.6%) obtained their prescriptions from traditional Chinese medicine doctors and 189 (30.8%) acquired TCHM from Chinese herbal stores. The common preparations were herbal decoction (42.5%), concentrated scientific Chinese medicine (33.8%), and traditional powder or pills (30.4%). Among all patients, women (59.9%) and those with a high household income (54.8%) tended to use TCHM (*p* values of <0.001 and <0.05, respectively), and women (74.3%) and those with a high education level (84.9%) and household income (77.4%) had an increased tendency to consume supplements (all *p* values < 0.001).

### 3.2. Discontinuation of Traditional Chinese Herbal Medicine or Supplements and Combined Conventional Western Medications

Table 2 presents the preoperative use of TCHM and supplements concurrently with conventional Western medications among participants. Among 727 patients taking TCHM, only 127 (17.5%) patients reported to discontinue using it before surgery and 78 (10.7%) patients reported to discontinue using it 4.7 ± 5.1 (1–24) days before the surgery. Furthermore, 47.3% of the 727 patients were diagnosed with at least one comorbidity, and up to 263 (36.2%) patients were taking TCHM and physician-prescribed Western medicine, such as antihypertensive medications (16.2%), analgesics (10.7%), or diabetes medications (6.9%), concurrently for their current diseases or underlying medical conditions. In addition, 359 (36.7%) of 977 patients were taking supplements and physician-prescribed Western medicine concurrently. Regarding underlying diseases, the use of TCHM and supplements was mostly reported by patients with asthma, kidney disease, liver disease, and hypertension before the surgery, and those undergoing gynecologic surgery, otorhinolaryngologic surgery, neurosurgery, general surgery, and orthopedic surgery.

### 3.3. Commonly Used Single and Compound Preparations of Traditional Chinese Herbal Medicine

Table 3 presents the top 10 single and compound herbal remedies used by 90% and 94% of the TCHM users, respectively. The most commonly used compound preparations among the participants were Si-Shen-Tang (consisting of four ingredients, including dried Chinese yam (*Dioscoreae rhizome*), lotus seed (*Nelumbo nucifera*), gorgon seed (*Euryale ferox*), and poria (*Pachyma hoelen*)) and Si-Wu-Tang (consisting of four ingredients, including Danggui (*Angelica sinensis*), Chuanxiong (*Ligusticum chuanxiong*), Chinese peony (*Paeonia lactiflora*), and cooked rehmannia (*Rehmannia glutinosa*)), which were used by 350 (48.1%) and 230 (31.6%) patients, respectively. Furthermore, the commonly used single herbs included goji berry (*Lycium barbarum*) (62.9%), jujube fruit (*Ziziphus jujuba*) (61.4%), Chinese yam (*Dioscoreae rhizome*) (60.3%), lotus seed (*Nelumbo nucifera*) (58.7%), and Danggui (*Angelica sinensis*) (54.3%), which are also popular food ingredients in Taiwan.

### 3.4. Commonly and Less Commonly Used Supplements

As summarized in Table 4, Taiwanese surgical patients did not commonly use supplements popular in Western societies, such as garlic (8.6%), ginkgo (6.5%), chamomile (5.5%), turmeric (4.6%), kava (0.0%), and St. John’s wort (0.0%).

## 4. Discussion

### 4.1. Major Finding

This study revealed a high prevalence of the presurgical use of TCHM and dietary supplements among Taiwanese surgical patients, particularly among those undergoing gynecologic and otorhinolaryngologic surgery. More than one-third of the patients reported concurrent use of herbal remedies or dietary supplements with prescribed Western medicine for their underlying diseases. Less than one-fifth of the patients who took Chinese herbal medicines reported to discontinue using them 4.7 ± 5.1 (1–24) days before the operation. Some commonly used herbal medicines in Taiwan may potentially modulate hepatic metabolization and increase bleeding risk during surgery. We suggest that surgeons and anesthesiologists should be informed of herbal medicine use in patients before admission, and they should be aware of the potential adverse effects of drug–herb interaction.

### 4.2. Current Recommendations for Preoperative Discontinuation of Herbal Medicines

Since 2001, concerns have been raised regarding anesthesia use in patients with presurgical use of herbal medicine [3,4,5,6,12]. Ang-Lee et al. listed eight commonly used herbal medicines and their perioperative concerns [3]. Varied durations for preoperative discontinuation of herbal medicines were recommended, for example, 24 h for kava, 36 h for ginkgo, 5 days for St John’s wort, and 7 days for garlic and ginseng [3]. In 2003, the American Society of Anesthesiologists issued the educational brochure, *Considerations for Anesthesiologists: What You Should Know About Your Patients’ Use of Herbal Medicines and Other Dietary Supplements* [21], which briefly described the trends of herbal medicine use, governmental oversight, potential side effects, and drug–herb interactions; however, it did not provide definite recommendations for the discontinuation of herbal medicine use before a surgery. Furthermore, the Second American Society of Regional Anesthesia and Pain Medicine Consensus Conference on Neuraxial Anesthesia and Antithrombotic Therapy in 2003 stated that the incidence rate of epidural or spinal hematoma after neuraxial anesthesia was not higher among patients who consumed herbal medicines than among patients who did not consume herbal medicines [22]. According to the patient educational brochure, *Herbal and Dietary Supplements and Anesthesia*, issued by the American Society of Anesthesiologists in 2015 [17], the sale of herbal or dietary supplements generates USD 27 billion a year, with half of the American population consuming them. The proportions of ingredients in each product are not closely inspected by the US Food and Drug Administration, and therefore, the consumption of these products can increase the risk of adverse effects during the surgery or procedure, including prolonged anesthesia effects, increased bleeding, increased blood pressure, interference with other medications, and heart problems. Patients must inform their anesthesiologists regarding their herbal medicine use during the pre-surgery visit and eventually, in some cases, may have to stop taking herbal and dietary supplements at least 2 weeks before a surgical procedure [17]. Currently, no official guidelines from the American Society of Anesthesiologists and the American College of Surgeons in the United States, Hong Kong, and Taiwan recommend the duration for which Western herbal or traditional Chinese herbal medicine use must be discontinued preoperatively.

### 4.3. Preoperative Use of Western and Chinese Herbal Medicines

The percentages of herbal medicine use among surgical patients before surgery were 3.6%–16% in Western societies [10,12,13,14] and 8%–13% in Hong Kong [15,16]. Furthermore, up to 80%–90% of the surveyed surgical patients in Hong Kong took self-prescribed traditional Chinese medicine on a daily basis in traditional soups and teas [15,16]. In Taiwan, traditional Chinese and Western medicines have been integrated in many conventional Western general hospitals and are covered by the Taiwan National Health Insurance [18]. In 2001, 28.4% of 21.6 million valid beneficiaries of the Taiwan National Health Insurance system used traditional Chinese medicine prescribed by traditional Chinese physicians [18]. A questionnaire survey conducted in 2018 revealed 37% and 37.3% of the 1200 respondents were prescribed or advised to use Chinese herbal medicine by Western medicine physicians and traditional Chinese medicine doctors, respectively [23]. Additionally, 5.2% of the Taiwanese population purchased traditional Chinese medicine without a physician’s prescription [19]. Among outpatients in a tertiary Western general hospital in Taiwan, Hong-Hwa (5.8%) and Jia-Wey-Shiau-Yau-San (3.8%) were the most commonly prescribed single and compound preparations, respectively, for the outpatients with the main diagnoses of insomnia (15.6%), menopause (5.2%), and constipation (5.1%) [20]. In the current study consisting of presurgical patients, goji berry (62.9%), jujube fruit (61.4%), Si-Shen-Tang (48.1%), and Si-Wu-Tang (31.6%) were the commonly used single and compound remedies, which are common ingredients of diet therapy in Chinese societies. The spectrum and use of herbal medicines before surgery in Taiwan are different from those in Western societies. We observed that Taiwanese surgical patients do not usually use supplements that are common in Western societies, such as ginkgo (6.5%), chamomile (5.5%), turmeric root (4.6%), kava (0.0%), and St. John’s wort (0.0%). Evidence suggests that goji berry (*Lycium barbarum*) has antioxidative, antiaging, immunoenhancing, and hypoglycemic properties [24]. Si-Shen-Tang, which consists of dried Chinese yam, lotus seed, gorgon seed, and poria, is the most famous herbal soup used in traditional Chinese medicine to nourish the digestive system. Jujube fruit and Si-Wu-Tang, which consists of *Angelica sinensis*, *Ligusticum chuanxiong*, *Paeonia lactiflora*, and *Rehmannia glutinosa*, strengthen the female reproductive system and have similar multiple effects on health promotion [25]. One-third of the presurgical patients in our study consumed herbal medicines or supplements concurrently with prescribed Western medicines. However, constituent pharmacokinetic interactions and toxicity risks of traditional Chinese remedies are limited [26,27,28], leading to a lack in definite discontinuation recommendations for compound herbal remedies before surgery.

### 4.4. Pharmacokinetic Consideration

Pharmacokinetic interactions of widely used herbal medicines have been screened for anti-inflammatory activity [26], inhibition of the intestinal cytochrome P450 3A4 enzyme activity [25], and drug metabolic enzymes and transporters [27]. Nevertheless, the extent to which individual ingredients are clinically active and their residual effects on the body are difficult to determine. For example, the pharmacokinetics of ginsenosides, based on its rapid elimination, with a half-life of 0.8–7.4 h in rabbits, suggests discontinuing ginseng use at least 24 h prior to surgery [3]. However, the discontinuation of ginseng use for at least 7 days is probably prudent, because ginseng irreversibly inhibits platelets [3]. Various reported herb–drug interactions have their pharmacokinetic bases, but their safety profiles are usually limited to animal studies and case reports. The ingredients of blended Chinese herbal medicines are too complicated to identify their additive or synergistic effects. A hospital-based survey in Taiwan revealed that two-thirds of the herbal prescriptions were compound formulae [19], and only 19% of the surgical patients in Hong Kong knew the composition of their Chinese herbal prescriptions [14]. Therefore, even surgeons and anesthesiologists are unable to identify the components of herbal medicine used by patients in the pre-surgery visit. Patients should be encouraged to obtain a copy of the prescription provided to them by the traditional Chinese medicine practitioner for future reference [15].

### 4.5. Perioperative Concerns and Predisposing Factors

Concerns regarding the interactions of herbal medications with anesthesia, particularly reduced coagulation, have increased in the past two decades [3]. Many commonly used Chinese herbs, such as ginseng, Danshen, Danggui, ginkgo, licorice, and turmeric, are known to increase bleeding risks due to their pharmacodynamic interactions with platelets and warfarin [29]. In a prospective survey in Hong Kong, surgical patients who took traditional Chinese medicine were likely to have a preoperative hypokalemia and prolonged international normalized ratio or activated partial thromboplastin time (adjusted relative risk, 2.21; 95% confidence interval, 1.14–4.29), however, without significant association with intraoperative or postoperative adverse events, compared with nonusers [16]. Another survey in Jamaica reported an increased blood loss (291.7 mL, *p* = 0.039) in surgical gynecologic patients with the recent use of certain botanicals with anticoagulant properties [11]. In the current study, TCHM use was common among Taiwanese patients undergoing gynecologic and otorhinolaryngologic procedures and among those with a diagnosis of asthma. Those with a higher household income or education level may consider the sources for acquiring and knowing these products, which may contribute to an increased tendency to consume TCHM or supplements in the population. Our results highlight the prevalence of TCHM use among these presurgical patients in Taiwan, indicating the need to educate the surgeons, anesthesiologists, and surgical patients in Taiwan regarding the predisposing factors and potential adverse effects of common traditional Chinese herbs.

### 4.6. Limitations

First, in this study, the high prevalence of preoperative herbal medicine use included prescribed, self-purchased, and dietary herbal remedies. Herbal diet therapy for health promotion and disorder prevention is a common concept in Chinese culture. We asked surgical patients to recall the herbal medicines used before hospital admission, but did not obtain information regarding the precise dosage, frequency, and duration of each medicine. Thus, recall bias may have occurred, which might have interfered with the results of the actual consumption and residual effects of the herbal medicines. Second, a mixture of ingredients in a compound formula and composition variations in a dosage or nonprescribed preparations considerably complicate potential adverse reactions. Some authorities recommended obtaining data regarding all herbal intake as part of the anesthetic assessment; however, this is difficult. Third, the patients in a medical center in Taipei might not represent the whole population in Taiwan. This study was designed to reveal the epidemiological data of TCHM use in Taiwanese surgical patients, rather than perioperative events related to herb–drug interaction. We therefore provide the predisposing factors for future countrywide studies to identify surgical patients who tend to take herbal medicines in Taiwan.

## 5. Conclusions

This questionnaire survey reveals the prevalence, predisposing factors, and discontinuation of presurgical use of TCHM and dietary supplements in Taiwanese surgical patients and encourages anesthesiologists and surgeons to inquire about TCHM use in patients during the preoperative assessment. Some commonly used herbal medicines in Taiwan may potentially modulate hepatic metabolization and increase bleeding risk during surgery. Furthermore, this questionnaire survey highlights the need for patient education materials regarding the safe use of concomitant Western medicine prescribed and the discontinuation of TCHM use before surgery in Taiwan.

## Figures and Tables

**Table 1:** Demographic data of presurgical patients who used traditional Chinese herbal medicine or supplements.

All, *n* = 1428	TCHM		Supplements	
	727 (50.9)	OR (95% CI)	977 (68.4)	OR (95% CI)
Sex				
Men, *n* = 805	354 (44.0)		514 (63.9)	
Women, *n* = 623	373 (59.9)	1.90 (1.54–2.35) ^a^	463 (74.3)	1.64 (1.30–2.06) ^a^
Age, year	44.5 ± 15.6		46.2 ± 15.7	
20–39, *n* = 537	291 (54.2)		354 (65.9)	
40–64, *n* = 703	346 (49.2)	0.82 (0.65–1.03)	485 (69.0)	1.15 (0.91–1.46)
≥65, *n* = 188	90 (47.9)	0.78 (0.56–1.08)	138 (73.4)	1.43 (0.99–2.07)
Education				
High school or less, *n* = 731	358 (49.0)		467 (63.9)	
University/college, *n* = 596	316 (53.0)	1.18 (0.95–1.46)	425 (71.3)	1.41 (1.11–1.77) ^b^
Graduate school, *n* = 93	48 (51.6)	1.11 (0.72–1.71)	79 (84.9)	3.19 (1.77–5.74) ^a^
Household income per month				
USD < 1000, *n* = 423	192 (45.4)		260 (61.5)	
USD 1000–3000, *n* = 629	329 (52.3)	1.32 (1.03–1.69) ^c^	441 (70.1)	1.47 (1.13–1.91) ^b^
USD > 3000, *n* = 230	126 (54.8)	1.46 (1.06–2.01) ^c^	178 (77.4)	2.15 (1.49–3.09) ^a^
Sources	*n* = 614		*n* = 657	
Prescription by traditional Chinese medicine doctor	329 (53.6)	-	-	-
Pharmacy	15 (2.4)	-	135 (20.5)	-
Chinese herbal medicine store	189 (30.8)	-	34 (5.2)	-
Friends and relatives	146 (23.8)	-	298 (45.4)	-
Direct selling	6 (1.0)	-	97 (14.8)	-
Media (radio, television)	5 (0.8)	-	17 (2.6)	-
Methods of preparation	*n* = 595		*n* = 793	
Chinese herb decoction	253 (42.5)	-	143 (18.0)	-
Concentrated scientific Chinese medicine	201 (33.8)	-	-	-
Traditional herbal powder or pills	181 (30.4)	-	-	-
Chinese medicinal cuisine	179 (30.1)	-	-	-
Biotechnology products	60 (10.1)	-	706 (89.0)	-

Data are presented as number (%) or mean ± standard deviation (range). TCHM, traditional Chinese herbal medicine; OR, odds ratios; CI, confidence interval. Odds ratios were estimated through unconditional logistic regression, with *p* values ^a^ < 0.001, ^b^ < 0.01, and ^c^ < 0.05, as compared with the first subgroup item.

**Table 2:** Preoperative use and discontinuation of traditional Chinese herbal medicine or dietary supplements and combined conventional Western medications.

	TCHM *n* = 727	Supplements *n* = 977
Number of items		
1–3	176 (24.5)	362 (37.1)
4–6	90 (12.4)	177 (18.1)
7–9	91 (12.5)	119 (12.2)
10–19	226 (31.1)	216 (22.1)
20–29	104 (14.3)	67 (6.9)
≥30	40 (5.5)	36 (3.7)
Discontinuation of herbal use before surgery, *n*	127 (17.5)	247 (25.3)
Days of discontinuation before surgery, day	4.7 ± 5.1 (1–24)	2.9 ± 2.1 (1–7)
Number of comorbidities		
0	383 (52.7)	500 (51.2)
1	257 (35.4)	347 (35.5)
2	58 (8.0)	83 (8.5)
≥3	29 (4.0)	47 (4.8)
Common comorbidities		
Asthma, *n* = 51	31 (60.8)	38 (74.5)
Kidney disease, *n* = 61	33 (54.1)	39 (63.9)
Liver disease, *n* = 59	30 (50.8)	42 (71.2)
Cancer, *n* = 183	91 (49.7)	128 (69.9)
Hypertension, *n* = 272	129 (47.4)	196 (72.1)
Diabetes, *n* = 115	49 (42.6)	66 (57.4)
Concurrent use of Western medications		
No Western medication	464 (63.8)	618 (63.3)
At least one medication	263 (36.2)	359 (36.7)
Antihypertensive medications	118 (16.2)	175 (17.9)
Analgesics	78 (10.7)	95 (9.7)
Diabetes medications	50 (6.9)	72 (7.4)
Cardiac medications	28 (3.9)	50 (5.1)
Steroids	17 (2.3)	22 (2.3)
Anticoagulants	14 (1.9)	28 (2.9)
Surgical procedures		
Gynecologic surgery, *n* = 105	72 (68.6)	76 (72.4)
Otorhinolaryngologic surgery, *n* = 187	105 (56.1)	135 (72.2)
Neurosurgery, *n* = 113	59 (52.2)	78 (69.0)
General surgery, *n* = 135	68 (50.4)	92 (68.1)
Orthopedic surgery, *n* = 478	239 (50.0)	334 (69.9)

Data are presented as number (%) or the mean ± standard deviation (range).

**Table 3:** Commonly used single and compound preparations of traditional Chinese herbal medicine in presurgical patients (*n* = 727).

Traditional Chinese Herbal Medicine	Frequent Use (%)	Occasional Use (%)	Sum (%)
Single herbal medicine			
1. Goji berry (*Lycium barbarum*)	56 (7.7)	401 (55.2)	457 (62.9)
2. Jujube fruit (*Ziziphus jujuba*)	52 (7.2)	394 (54.2)	446 (61.4)
3. Chinese yam (*Dioscoreae rhizome*)	58 (8.0)	380 (52.3)	438 (60.3)
4. Lotus seed (*Nelumbo nucifera*)	33 (4.5)	394 (54.2)	427 (58.7)
5. Danggui (*Angelica sinensis*)	13 (1.8)	382 (52.5)	395 (54.3)
6. Dried longan pulp (*Dimocarpus longan*)	18 (2.5)	336 (46.2)	354 (48.7)
7. Korean ginseng (*Panax ginseng*)	23 (3.2)	313 (43.1)	336 (46.3)
8. Niu-Bong (*Arctium lappa*)	24 (3.3)	305 (42.0)	329 (45.3)
9. Huang-Qi (*Astragalus membranaceus*)	16 (2.2)	278 (38.2)	294 (40.4)
10. Chrysanthemum flower	22 (3.0)	250 (34.4)	272 (37.4)
Compound preparation			
1. Si-Shen-Tang	14 (1.9)	336 (46.2)	350 (48.1)
2. Si-Wu-Tang	9 (1.2)	221 (30.4)	230 (31.6)
3. Shih-Chuan-Da-Bu-Tang	4 (0.6)	143 (19.7)	147 (20.3)
4. Zhi-Sou-San	3 (0.4)	123 (16.9)	126 (17.3)
5. Danggui-Buxue-Tang	1 (0.1)	125 (17.2)	126 (17.3)
6. Bu-Yao-Jiu	3 (0.4)	83 (11.4)	86 (11.8)
7. Yun-Gong-San	0 (0.0)	70 (9.6)	70 (9.6)
8. Huang-Lien-Gieh-Du-Tang	3 (0.4)	62 (8.5)	65 (8.9)
9. Sheng-Hua-Tang	2 (0.3)	55 (7.6)	57 (7.8)
10. Gui-Lu-Er-Xian-Jiao	1 (0.1)	48 (6.6)	49 (6.7)

Data are presented as number (%).

**Table 4:** Commonly and less commonly used supplements in presurgical patients (*n* = 977).

Supplements	Frequent Use (%)	Occasional Use (%)	Sum (%)
Common use			
1. Vitamins	283 (29.0)	309 (31.6)	592 (60.6)
2. Green tea (soft drink)	145 (14.8)	238 (24.4)	383 (39.2)
3. Cranberry juice	40 (4.1)	317 (32.4)	357 (36.4)
4. Honey	48 (4.9)	301 (30.8)	349 (35.7)
5. Calcium supplement	100 (10.2)	233 (23.8)	333 (33.8)
6. Onion	102 (10.4)	206 (21.1)	308 (31.5)
7. Vitamin E	65 (6.7)	234 (24.0)	299 (30.7)
8. Glucosamine	139 (14.2)	159 (16.3)	298 (30.5)
9. Ginger	87 (8.9)	197 (20.2)	284 (29.1)
10. Grapefruit juice	22 (2.3)	245 (25.1)	267 (27.4)
Less common use			
Shiitake mushroom	81 (8.3)	142 (14.5)	223 (22.8)
Reishi mushroom (Ling-Zhi)	13 (1.3)	90 (9.2)	103 (10.5)
Monascus	8 (0.8)	159 (16.3)	167 (17.1)
Garlic	8 (0.8)	76 (7.8)	84 (8.6)
Ginkgo	12 (1.2)	52 (5.3)	64 (6.5)
Chamomile	1 (0.1)	53 (5.4)	54 (5.5)
Turmeric	8 (0.8)	37 (3.8)	45 (4.6)
Kava	0 (0.0)	0 (0.0)	0 (0.0)
St. John’s wort	0 (0.0)	0 (0.0)	0 (0.0)

Data are presented as number (%).

## Data Availability

The data presented in this study are available upon request from the corresponding authors.
